# Stenosing Ileal Angiosarcoma Presenting as Subocclusive Syndrome: A Rare Case Report and Literature Review

**DOI:** 10.3390/diagnostics16142245

**Published:** 2026-07-18

**Authors:** Mircea Vasile Milaciu, Cezara Andreea Gerdanovics, Olga Hilda Orășan, Angela Cozma, Teodora Gabriela Alexescu, Călin Vasile Vlad, Anamaria Vlăduța Tomoiagă, Vlad Alexandru Zolog, Vlad Dumitru Brata, Lorena Ciumărnean, Nicoleta Valentina Leach, Flaviu Mureșan, Codruța Claudia Gherman Lencu, Adriana Zolog, Anca Mihailov, Mirela Georgiana Perne

**Affiliations:** 1 Department of Internal Medicine, 4th Medical Discipline, ”Iuliu Hațieganu” University of Medicine and Pharmacy, Republicii Street, No. 18, 400015 Cluj-Napoca, Romaniateodora.alexescu@umfcluj.ro (T.G.A.); vladutat@yahoo.com (A.V.T.);; 2 Department of Gastroenterology, Regional Institute of Gastroenterology and Hepatology “Prof. Dr. Octavian Fodor”, 400394 Cluj-Napoca, Romania; 3 2nd Department, Faculty of Nursing and Health Sciences, ”Iuliu Hațieganu” University of Medicine and Pharmacy, Republicii Street, No. 18, 400015 Cluj-Napoca, Romanianicoleta_leach@yahoo.com (N.V.L.); 4 4th Surgical Department, “Iuliu Hațieganu” University of Medicine and Pharmacy, Republicii Street, No. 18, 400015 Cluj-Napoca, Romania; 5 Department 1, Fundamental Discipline, Endocrinology, “Iuliu Hațieganu” University of Medicine and Pharmacy, Victor Babeș Street nr. 8, 400347 Cluj-Napoca, Romania; codruta_lmc@yahoo.com; 6 Clinical Hospital CF Cluj-Napoca, Republicii Street, No. 18, 400015 Cluj-Napoca, Romania; adizolog@yahoo.com (A.Z.);

**Keywords:** angiosarcoma, ileal angiosarcoma, primary gastrointestinal angiosarcoma, small-bowel tumor, Crohn’s disease mimic, intestinal stenosis

## Abstract

**Introduction:** Angiosarcomas are rare malignant vascular tumors. Primary angiosarcoma of the ileum is extremely uncommon, with few cases reported in the literature. **Case Report:** We report the case of a 54-year-old woman who presented with abdominal pain and constipation. Endoscopic evaluation did not reveal a mass in the distal ileum; however, MRI identified a distal ileal stenosis, raising suspicion for Crohn’s disease. Surgical resection was performed, and histopathological analysis confirmed the diagnosis of primary ileal angiosarcoma. Postoperative chemotherapy was attempted but not tolerated, and the patient subsequently developed suspected peritoneal progression approximately six months after surgery. **Discussion:** Due to its rarity and nonspecific symptoms, ileal angiosarcoma is often diagnosed late. Imaging may suggest a vascular tumor, but definitive diagnosis requires histopathological and immunohistochemical analysis. Prognosis is generally poor, with high metastatic potential. **Conclusions:** This case highlights the diagnostic challenges of ileal angiosarcoma, a very rare type of tumor of the small bowel.

## 1. Introduction

Angiosarcoma is a rare and highly aggressive malignant neoplasm arising from vascular or lymphatic endothelial cells, accounting for only 1–2% of all soft tissue sarcomas [1,2]. Although these tumors most commonly develop in the skin and soft tissues, the involvement of visceral organs is uncommon, and primary gastrointestinal angiosarcoma represents an exceedingly rare entity [3,4,5]. Current knowledge is largely based on isolated case reports and small literature reviews. Primary angiosarcoma of the small intestine is even rarer, with only a limited number of cases reported in the English-language literature [6,7,8]. Its early clinical presentation is usually nonspecific, which may obscure clinical suspicion and delay diagnosis. Reported symptoms include abdominal pain, weight loss, nausea, vomiting, gastrointestinal bleeding, and, less commonly, obstructive or subocclusive manifestations [9]. Several risk factors have been implicated in the development of angiosarcoma, including previous trauma, exposure to vinyl chloride, and radiotherapy. Nevertheless, the occurrence of angiosarcoma after exposure to these factors remains rare; for example, the overall risk of post-radiotherapy angiosarcoma has been estimated to range between 0.01% and 0.30% [10].

The timely diagnosis of small intestinal angiosarcoma remains difficult because of its rarity, heterogeneous and nonspecific clinical manifestations, and the limited sensitivity of conventional diagnostic methods, resulting in delayed diagnosis and poor prognosis [3]. Imaging findings may suggest an inflammatory, stenosing, or neoplastic process, but definitive diagnosis relies on histopathological and immunohistochemical examination. Due to the scarcity of cases, there are no standardized therapeutic guidelines. Surgical resection remains the mainstay of treatment, occasionally combined with adjuvant therapy [11,12]. Despite treatment, the prognosis is generally poor, reflecting the aggressive biological behavior of this malignancy, the frequent delay in diagnosis, and the lack of sufficient evidence guiding therapeutic management [13,14].

The aim of this case report is to describe a rare case of stenosing ileal angiosarcoma presenting with subocclusive symptoms in a patient with a history of pelvic radiotherapy for cervical cancer. By integrating the clinical course with a review of the available literature, we aim to highlight the diagnostic challenges, histopathological and immunohistochemical features, therapeutic limitations, and poor prognosis associated with this rare small intestinal malignancy.

## 2. Case Presentation

We present the case of a 54-year-old woman with a history of surgically treated and chemo-radiotreated cervical squamous cell carcinoma and suspected postoperative adhesive syndrome, who developed progressive abdominal symptoms. Her past medical history was notable for cervical squamous cell carcinoma, staged pT1bN1L1V1Pn1, treated in 2016 by Wertheim-type surgery, chemotherapy, and external pelvic radiotherapy. The available documentation also mentioned a suspected adhesive syndrome without a previously established definitive diagnosis. Family history was unremarkable. The patient denied smoking, alcohol use, relevant toxic exposures, and chronic home medication. The current disease began insidiously in January 2024, with episodic constipation and diffuse abdominal pain, initially interpreted in the context of irritable bowel syndrome with constipation predominance and possible postoperative adhesions. Over the following months, the symptoms progressed slowly, with diffuse colicky abdominal pain, predominantly peri-umbilical and in the right abdominal flank; nausea; alimentary and bilio-enteric vomiting; reduced tolerance of some solid foods; palpitations; exertional asthenia; fatigability and a 13 kg weight loss over approximately two months.

The patient presented to the Internal Medicine Department of the Clinical CF Hospital in Cluj-Napoca in June 2024 for progressive abdominal pain, constipation, nausea/vomiting and weight loss. On admission, the patient was afebrile, with mildly impaired general condition, pale and dehydrated mucosae, and grade I obesity. Abdominal examination showed no signs of acute abdomen and no palpable tumor masses. Neurological examination was unremarkable. Initial laboratory evaluation showed a mildly inflammatory profile, with ESR 23 mm/h and CRP 7.97 mg/L, mild hypercholesterolemia and exclusion of hypothyroidism.

Abdominal ultrasonography showed hepatic steatosis with areas of focal sparing and postoperative hysterectomy status. Contrast-enhanced abdomino-pelvic CT did not identify hepatic nodular lesions but described, in the pelvis, a dilated intestinal loop with liquid content (Figure 1A). Colonoscopy was initially postponed at the patient’s request.

As symptoms progressed, repeat biological evaluation showed ESR 34 mm/h, CRP 33.76 mg/L, a mild nitrogen retention syndrome, and new mild normocytic anemia (hemoglobin 11.2 g/dL; hematocrit 34.9%). Upper digestive endoscopy identified a gastric phytobezoar (Figure 2). Ileocolonoscopy showed no lesions in the terminal ileum over the examined 10 cm, and the cecum, ileocecal valve, ascending colon, sigmoid colon, rectum, and anal canal were without relevant lesions, apart from congested grade II internal hemorrhoids.

Further imaging by native abdomino-pelvic CT and enterographic MRI (Figure 1B,C) identified dilated small-bowel loops with liquid content and a circumferential, gadolinium-enhancing parietal thickening in the right parasagittal pelvic region, associated with stenosis and the upstream dilation of small-bowel loops. These findings raised the suspicion of stenosing ileal Crohn’s disease. The associated diagnoses included subocclusive syndrome (König syndrome), gastric phytobezoar identified on upper digestive endoscopy, postoperative adhesive syndrome after surgery and chemo-radiotherapy for cervical cancer, mild normochromic normocytic hyposideremic anemia, and obesity grade I.

The patient was prepared for surgery with dietary measures, hydro-electrolytic rebalancing, nutritional support, intravenous albumin, antibiotic preparation, gastric antisecretory therapy, analgesics, antiemetics, and antispasmodics. During the first surgical intervention, performed under general anesthesia with oro-tracheal intubation through supra- and infra-umbilical median laparotomy, a submesocolic adhesive syndrome was found. Jejunal and ileal loops were distended, thickened, edematous and filled with fluid. A stenosis was identified at the level of the last ileal loop, followed by approximately 20 cm of the ileum with apparently normal caliber and appearance. The stenotic loop was adherent to the posterior pelvic wall and angulated. No intra-abdominal lesions suspicious for recurrent neoplasia were detected. Adhesiolysis and segmental ileal enterectomy of approximately 20 cm with latero-lateral entero-enteral anastomosis and Douglas drainage were performed.

The early postoperative course was complicated by the failure of bowel transit to resume despite multiple prokinetic and pharmacologic approaches, bilious vomiting, and ultrasound findings of dilated small-bowel loops with fluid content and mixing movements, without intra-abdominal collections. Postoperative monitoring showed large gastric aspirate volumes, mild renal dysfunction/nitrogen retention, and initial hypokalemia. Abdomino-pelvic CT (Figure 1D) on postoperative day 4 described the collapse of the ileal segment distal to the latero-lateral anastomosis, dilation of the upstream small bowel with fluid content, a collapsed colon, and no intra-abdominal collections.

Because of postoperative intestinal obstruction due to stenosis of the latero-lateral ileo-ileal anastomosis, the patient underwent reintervention on postoperative day 9. Intraoperatively, the bowel loops upstream of the anastomosis were distended and edematous, the ileo-ileal latero-lateral anastomosis was stenotic and an ileocecal inflammatory block was present, without intra-abdominal collections. A latero-lateral ileocecal anastomosis as an internal derivation was performed, with drainage. After reintervention, the nasogastric tube was suppressed after 24 h, with intestinal transit resumed for gas on day 2 after enema, but from day 3, the patient developed diarrhea. Repeated testing for Clostridioides difficile was negative, and stool culture/PCR testing was negative for Salmonella, Shigella, *Escherichia coli*, *Staphylococcus aureus*, *Bacillus cereus* and *Candida* spp. Symptoms improved under constipating diet, diosmectite, loperamide and supportive therapy.

Histopathological examination of the resected ileal segment (Figure 2A–H) revealed a stenosing malignant vascular tumor measuring approximately 6 cm, associated with mucosal ulceration and infiltration of the intestinal wall. Microscopically, the tumor was composed of epithelioid and elongated atypical cells with indistinct cellular borders, moderately eosinophilic cytoplasm, round-to-oval nuclei with vesicular chromatin, and rare visible nucleoli. The neoplastic proliferation showed a diffuse and irregular infiltrative pattern, involving the lamina propria, submucosa, muscularis propria, and focally the subserosal layer, with focal tumor necrosis. The mitotic count was 5 mitoses/10 high-power fields, and the Ki-67 proliferation index was approximately 33%. No lymphatic or venous tumor emboli were identified (L0/V0), and no perineural invasion was observed (Pn0). The proximal, distal, and radial resection margins were free of tumor, consistent with R0 resection. The tumor was classified as FNCLCC grade II, pT1 pN0 pMx.

Immunohistochemical examination supported endothelial differentiation, with diffuse intense positivity for CD31 and ERG and partial positivity for CD34 in the tumor cells. c-KIT and DOG1 were negative, arguing against gastrointestinal stromal tumor. Additional markers, including SMA, SOX10, CKAE1/AE3, and p63, were negative in the tumor cells. Overall, the morphological and immunohistochemical findings supported the diagnosis of primary stenosing ileal angiosarcoma.

The final positive diagnosis was stenosing ileal angiosarcoma, together with submesocolic adhesive syndrome after Wertheim surgery and external radiotherapy for cervical cancer, postoperative intestinal obstruction due to stenosis of the entero-enteral anastomosis with ileocecal inflammatory block, metabolic dysfunction-associated steatohepatitis, moderate chronic anemia, hypoproteinemia and obesity grade I.

Postoperative oncologic treatment was attempted twice in the patient’s local center, but chemotherapy was not tolerated because of profound general deterioration up to circulatory shock, hematologic toxicity and severe sepsis. In November–December 2024, the patient had two episodes of urinary-source sepsis, treated locally. At the three-month follow-up in December 2024, the patient had persistent anemia and mild renal dysfunction, while abdomino-pelvic ultrasound showed no suspicious hepatic nodules, ascites, or retroperitoneal lymphadenopathy.

In February 2025, the patient’s condition deteriorated clinically and biologically, with massive serohemorrhagic ascites, progressive anemia, inflammatory syndrome, and the need for repeated transfusions. Native abdominal CT could not definitively confirm tumor recurrence but described postoperative changes, micronodular mesenteric lesions, omental infiltration, and massive ascites, raising suspicion of peritoneal disease progression. Paracentesis yielded serohemorrhagic ascitic fluid. Cytological and cytoblock examination did not identify malignant cells; therefore, recurrence was not histologically or cytologically confirmed.

Cytoblock examination did not identify malignant cells and showed normal and dysmorphic erythrocytes, hemosiderin and isolated mesothelial cells on a hemorrhagic background.

Laboratory evaluation demonstrated an inflammatory syndrome, progressive anemia, transient nitrogen retention/renal dysfunction and postoperative electrolyte disturbances. Values not present in the provided documents are marked as not reported.

The clinical course remained progressively unfavorable, with multiple organ failure, and the patient died on 5 March 2025.

This case illustrates the diagnostic difficulty of primary ileal angiosarcoma, which may mimic functional bowel disease or stenosing Crohn’s disease and may present as subocclusive syndrome. In patients with previous pelvic radiotherapy and progressive obstructive symptoms, rare small-bowel malignancies, including angiosarcoma, should be considered when imaging and endoscopy are discordant and symptoms progress despite conservative management.

## 3. Discussion

### 3.1. Rarity and Epidemiological Context of Small Intestinal Angiosarcoma

Angiosarcomas are highly malignant mesenchymal neoplasms with vascular or lymphatic endothelial differentiation, accounting for less than 2% of all soft tissue sarcomas [15,16]. Primary gastrointestinal angiosarcoma is even rarer, and current knowledge remains largely based on isolated case reports and small literature reviews [17,18,19,20,21,22].

Among primary small intestinal angiosarcomas, the jejunum and ileum are the most frequently reported sites. Therefore, the ileal location observed in our patient is consistent with previously reported small-bowel cases, although the tumor remains extremely rare in absolute terms [9]. Previous historical series and more recent reviews have consistently emphasized the exceptional rarity of gastrointestinal angiosarcoma and the difficulty of drawing firm conclusions regarding optimal diagnosis, treatment, and prognosis [23,24,25,26]. Although metastatic patterns vary across reported PGAS locations, distant disease is common overall in published reviews, with the liver, lungs, peritoneum, spleen, bone, stomach and mesentery among the reported metastatic sites [9]. However, larger pooled reviews indicate that distant disease is common overall. In one review, Nai et al. [9] found that multifocal PGAS was associated with distant metastases at diagnosis in 6 out of 10 patients, while small intestinal and colorectal PGASs presented with distant metastases in 44.9% and 53.8% of included cases, respectively. Reported metastatic sites include the liver and lungs, each in 42.3% of metastatic cases, followed by the peritoneum in 19.2%; spleen in 15.4%; and bone, stomach, and mesentery in 11.5% each, as well as less frequent involvement of the kidney, abdominal wall, retroperitoneum, brain, pleura, pancreas, omentum, gallbladder, urinary bladder, appendix, and skin [9].

Compared with these literature data, our case initially appeared localized, with no suspicious hepatic nodules, ascites, or retroperitoneal lymphadenopathy at early follow-up. However, the subsequent rapid deterioration, massive serohemorrhagic ascites, omental infiltration, micronodular mesenteric lesions, progressive anemia, inflammatory syndrome, and multiple organ failure were clinically and radiologically suggestive of aggressive disease progression. Because ascitic cytology was negative, this late evolution was considered suspected peritoneal progression rather than histologically confirmed recurrence. Overall, the present case is consistent with the literature regarding ileal localization, nonspecific presentation, aggressive evolution, and poor prognosis. Its distinctive features are the female sex, relatively young age, Crohn-like stenosing presentation, and previous pelvic radiotherapy, which should be interpreted as a possible etiological clue rather than definitive proof of causality. Table 1 summarizes selected previously reported ileal or terminal ileal angiosarcoma cases and highlights the main similarities with the present case, including nonspecific abdominal symptoms, frequent prior pelvic radiotherapy, diagnostic difficulty, surgical confirmation, and poor prognosis.

### 3.2. Nonspecific Clinical Presentation and Diagnostic Delay—Ileal Angiosarcoma Mimicking Stenosing Crohn’s Disease and Adhesive Disease

The clinical presentation of primary gastrointestinal angiosarcoma (PGAS) largely depends on tumor location and is usually nonspecific, which frequently delays clinical suspicion and diagnosis [32]. The most commonly reported manifestations include gastrointestinal bleeding, abdominal or anal pain, and obstructive symptoms [33]. Other possible features include nausea, vomiting, anemia, weight loss, diarrhea, and, less frequently, the detection of a palpable abdominal mass [34,35]. In the study by Schizas et al. [3], gastrointestinal bleeding and abdominal pain were observed in the majority of patients with small intestinal and colonic angiosarcomas, whereas dysphagia and epigastric pain were more typical of esophageal and gastric angiosarcomas, respectively. Melena has also been described as an unfavorable clinical indicator in small intestinal vascular tumors. Overall, the mean interval between symptom onset and diagnosis has been estimated to exceed six months, reflecting the difficulty of recognizing this rare entity in routine clinical practice [36]. In the present case, the diagnostic delay was multifactorial. In addition to the nonspecific clinical presentation and radiological overlap with more common conditions, colonoscopy was initially postponed at the patient’s request, and no preoperative histological sample could be obtained from the stenosing ileal lesion. Although ileocolonoscopy was subsequently performed, the examined terminal ileum showed no mucosal lesions, limiting the possibility of endoscopic biopsy. Therefore, the final diagnosis could only be established after surgical resection and histopathological/immunohistochemical examination of the ileal specimen.

This diagnostic delay is mainly explained by the overlap between the symptoms of small intestinal angiosarcoma and those of more common gastrointestinal disorders, including inflammatory bowel disease, gastrointestinal tumors, peptic ulcer disease, adhesive disease, and functional bowel disorders. In particular, abdominal pain, altered bowel habits, nausea, vomiting, weight loss, and anemia are not specific enough to immediately suggest a malignant vascular tumor. Therefore, angiosarcoma may only become suspected after complications occur or after surgical exploration and histopathological examination. In patients with unexplained gastrointestinal bleeding, especially when conventional endoscopic assessment is inconclusive, angiosarcoma should be included among the rare but aggressive differential diagnoses [3].

Small intestinal angiosarcoma may also present with abdominal complications such as obstruction, perforation, intussusception, or intraperitoneal hemorrhage, sometimes requiring urgent surgical management [10]. In contrast to cases dominated by overt gastrointestinal bleeding or perforation, our patient presented predominantly with progressive subocclusive symptoms, which contributed to the initial consideration of postoperative adhesive disease and stenosing Crohn’s disease. From an imaging perspective, the suspicion of stenosing Crohn’s disease was understandable, as the patient presented with circumferential ileal wall thickening, gadolinium enhancement, luminal narrowing, and upstream small-bowel dilation. These findings may overlap with fibrostenotic or inflammatory Crohn’s disease, particularly when located in the distal ileum. However, several elements were atypical for a straightforward diagnosis of Crohn’s disease. The patient had no previous history of inflammatory bowel disease, ileocolonoscopy did not reveal mucosal lesions in the examined terminal ileum, and the clinical course was characterized by progressive obstructive symptoms, marked weight loss, anemia, and inflammatory syndrome despite the absence of endoscopic confirmation. Moreover, the focal stenosing lesion occurred in a patient with previous pelvic surgery and radiotherapy, further complicating the differential diagnosis by also supporting postoperative adhesions or radiation-related bowel injury. In retrospect, the discordance between entero-MRI findings and ileocolonoscopy, together with the progressive clinical deterioration, should raise suspicion for an alternative diagnosis, including a rare small-bowel malignancy.

In our patient, several features gradually argued against a purely functional or benign adhesive process, including progressive symptom worsening, marked weight loss of 13 kg over two months, inflammatory syndrome, development of normocytic anemia, persistent obstructive manifestations and discordance between endoscopic and radiological findings.

Nevertheless, because primary ileal angiosarcoma is exceptionally rare and the ileal lesion was not accessible to preoperative biopsy, the definitive diagnosis could only be established after surgical resection and histopathological/immunohistochemical examination.

### 3.3. Previous Pelvic Radiotherapy as a Possible Etiological Clue

Several risk factors have been associated with the development of angiosarcoma, among which previous radiotherapy and chronic lymphedema, classically described as Stewart–Treves syndrome, are the most frequently recognized [37]. In order to classify a vascular malignancy as radiation-associated, the tumor should fulfill Cahan’s criteria, which generally require that the neoplasm arises within a previously irradiated field, appears after an adequate latency period, and is histologically distinct from the primary tumor for which radiotherapy was administered [38]. In primary gastrointestinal angiosarcoma, the estimated mean interval between radiation exposure and tumor development is approximately 12.5 years, while reported radiation doses generally range between 40 and 80 Gy [39].

Other etiological factors have also been implicated, although less consistently. Endothelial tumors have occasionally been described in association with foreign bodies, prior chemotherapy, and exogenous toxins, including vinyl chloride, thorotrast, and arsenic [35]. Familial or genetic syndromes, such as neurofibromatosis type 1 and Maffucci syndrome, as well as chronic inflammatory states, anal abscesses, fistulas, and peritoneal dialysis, have also been proposed as possible contributing factors [26,40]. Nevertheless, in many cases, no clear causative factor can be identified.

Most documented cases of post-radiation angiosarcoma involve the skin and underlying soft tissues, particularly after treatment for breast carcinoma. In contrast, gastrointestinal post-radiation angiosarcoma is exceptionally rare. Previously reported cases occurred after radiotherapy for several malignancies, including Hodgkin lymphoma, chondrosarcoma, endometrial carcinoma, cervical carcinoma, uterine leiomyosarcoma, ovarian malignancies, and other pelvic tumors [6,41]. The present case is relevant in this context because the patient had a history of cervical squamous cell carcinoma treated by surgery, chemotherapy, and external pelvic radiotherapy in 2016, followed by the development of ileal angiosarcoma in 2024, approximately eight years later. Although this latency interval may be compatible with a possible radiation-associated process, the available data are insufficient to establish a definitive causal relationship. Detailed information regarding the previous radiotherapy field, total dose, fractionation schedule, and anatomical overlap between the irradiated volume and the subsequent ileal tumor site was not available. Therefore, previous pelvic radiotherapy should be interpreted as a clinically relevant etiological clue and a possible contributing factor, rather than definitive evidence of radiation-induced angiosarcoma. This distinction is important because classification as a radiation-associated sarcoma generally requires the fulfillment of established criteria, including development within a previously irradiated field after an adequate latency period and histological distinction from the primary malignancy. This aspect adds an important learning point to the case. In long-term survivors of gynecological malignancies treated with pelvic radiotherapy, progressive obstructive small-bowel symptoms should not be attributed automatically to postoperative adhesions, radiation enteritis or functional bowel disease.

MYC assessment may provide additional support in the evaluation of suspected radiation-associated angiosarcoma. MYC amplification detected by fluorescence in situ hybridization or nuclear MYC overexpression by immunohistochemistry have been reported in many radiation-associated angiosarcomas and may help distinguish these tumors from benign or atypical post-radiation vascular proliferations. In the present case, MYC testing was not available. Therefore, the possible relationship with previous pelvic radiotherapy remains unconfirmed and should be interpreted cautiously.

Although exceedingly rare, intestinal angiosarcoma, including a possible radiation-associated form, should be considered in the differential diagnosis, particularly when symptoms are progressive, imaging demonstrates a focal stenosing lesion, and endoscopic findings are inconclusive.

### 3.4. Histopathological and Immunohistochemical Confirmation

The diagnosis of primary gastrointestinal angiosarcoma relies on the integration of clinical assessment, imaging, endoscopic findings, surgical exploration, and, ultimately, histopathological and immunohistochemical confirmation [3]. Conventional imaging modalities, including CT and MRI, have limited diagnostic specificity and are generally used to localize the lesion, assess complications, and evaluate possible metastatic spread [37,39]. CT findings may suggest a poorly defined mass, sometimes associated with increased density and intratumoral hemorrhage [42]. PET-CT may be useful for staging, assessment of metastatic disease, and monitoring response to treatment [43]. However, imaging alone is rarely sufficient for diagnosis, especially in small intestinal angiosarcoma, where radiological findings may overlap with inflammatory, ischemic, adhesive, or other neoplastic processes.

Endoscopy plays an important diagnostic role in esophageal, gastric, and colonic angiosarcomas, but its utility is more limited in small intestinal tumors because of the anatomical inaccessibility of many lesions. Endoscopically, primary gastrointestinal angiosarcoma may appear as a firm mass with hypervascular, hemorrhagic, ulcerated, or even apparently normal mucosa [33]. Capsule endoscopy and device-assisted enteroscopy may provide additional diagnostic information in selected cases of suspected small-bowel angiosarcoma [44]. Nevertheless, exploratory surgery remains a frequent diagnostic and therapeutic step in small intestinal cases, particularly when patients present with obstruction, perforation, bleeding, or an unclear stenosing lesion.

In the present case, the diagnostic pathway illustrates these limitations. Upper digestive endoscopy revealed a gastric phytobezoar, while ileocolonoscopy did not identify lesions in the examined terminal ileum. Entero-MRI suggested a stenosing ileal inflammatory process, initially raising suspicion of stenosing Crohn’s disease. The definitive diagnosis was established only after surgical resection of the stenotic ileal segment and histopathological examination.

Microscopically, well-differentiated gastrointestinal angiosarcoma is typically composed of anastomosing vascular channels lined by atypical endothelial cells, with variable intraluminal red blood cells. Poorly differentiated tumors may show solid sheets of atypical cells, necrosis, hemorrhage, marked cytologic atypia, and increased mitotic activity [3]. Epithelioid morphology may also be encountered [45]. In the present case, histopathological examination of the resected ileal segment confirmed a stenosing grade II FNCLCC ileal angiosarcoma, staged pT1N0Mx, complicated by intestinal subocclusion. The pathological slides also documented tumoral necrosis and mitotic activity, supporting the malignant vascular nature of the lesion.

Immunohistochemistry is essential both for confirming endothelial differentiation and for excluding other more common small intestinal tumors. CD31, CD34, factor VIII-related antigen, and vimentin are commonly reported positive markers in gastrointestinal angiosarcoma, while other endothelial markers such as von Willebrand factor, FLI-1, VEGF, UEA, and endothelin-1-like markers may also support the diagnosis, although with variable sensitivity [34]. In the present case, the immunohistochemical profile included endothelial markers such as CD31, CD34, and ERG, supporting vascular endothelial differentiation. Additional markers, including c-KIT and DOG1, were useful in the differential diagnosis, particularly for excluding gastrointestinal stromal tumor. Therefore, the combination of morphology and endothelial immunophenotype established the diagnosis of ileal angiosarcoma. At disease progression, the patient developed massive serohemorrhagic ascites, omental infiltration, micronodular mesenteric lesions, progressive anemia, inflammatory syndrome and clinical deterioration, raising suspicion of tumor recurrence and peritoneal involvement. Cytological and cytoblock examination of the ascitic fluid did not identify malignant cells. The cytoblock sections contained erythrocytes with usual or dystrophic morphology, a few lymphocytes, and small hemosiderin deposits, while the examined smears showed rare isolated mesothelial cells on a hemorrhagic background. Although these findings did not provide cytological confirmation of malignant ascites, they were consistent with hemorrhagic ascites and did not exclude peritoneal tumor progression. In this context, the subsequent clinical and radiological evolution remained highly suggestive of aggressive malignant recurrence. Although ascitic fluid cytology was negative for malignant cells, this result should be interpreted cautiously, as negative cytology does not exclude peritoneal involvement, particularly in the setting of hemorrhagic ascites, omental infiltration, mesenteric micronodular lesions and rapidly progressive clinical deterioration.

### 3.5. Therapeutic Challenges and Lack of Standardized Management

The classification of primary gastrointestinal angiosarcoma (PGAS) generally follows the staging system used for soft tissue sarcomas, combining TNM staging with histological tumor grade. PGASs are usually regarded as high-grade malignancies, reflecting their aggressive biological behavior and poor clinical outcomes [3]. In localized disease, surgical resection remains the preferred therapeutic approach and is often the only potentially curative option [7]. Although surgery with radical lymphadenectomy is considered standard in several soft tissue sarcomas, its value in angiosarcoma, and particularly in PGAS, remains uncertain [46,47]. This is partly explained by the rarity of the disease, the limited available evidence, and the frequent presence of multifocal, infiltrative, or metastatic disease at diagnosis.

In cases of disseminated angiosarcomatosis, complete R0 resection is often impossible, and compromised surgical margins are associated with poor prognosis [9]. Moreover, the prognostic impact of metastatic disease in PGAS remains difficult to define because most available data are derived from isolated case reports or small pooled analyses. Some reviews have suggested that metastatic disease may not significantly influence overall survival, but these observations should be interpreted cautiously due to the very limited number of cases and the heterogeneity of tumor sites, treatment strategies, and follow-up duration.

The role of adjuvant chemotherapy and radiotherapy remains undefined [48]. Several chemotherapeutic regimens have been used in PGAS, including paclitaxel, doxorubicin, vincristine, dacarbazine, and cyclophosphamide [6]. However, these approaches are largely extrapolated from the management of cutaneous and soft tissue angiosarcomas rather than being based on PGAS-specific evidence [6]. Chemotherapy appears particularly relevant in metastatic or unresectable disease, where it may improve progression-free survival [9]. In the systematic review by Schizas et al. [3], chemotherapy seemed to improve overall survival in patients with small intestinal angiosarcoma, whereas surgery appeared to have a more evident prognostic benefit in colonic angiosarcoma. Although neoadjuvant therapy was not reported in the included cases, Nai et al. proposed that neoadjuvant chemotherapy may be considered in selected patients with good performance status [9].

Radiotherapy has a controversial role in PGAS. On the one hand, it may be considered in selected unresectable or incompletely resected tumors, as in other sarcomas. On the other hand, previous radiotherapy is also a recognized risk factor for the development of secondary angiosarcoma, which limits enthusiasm for its use in certain clinical contexts [44]. In recent years, targeted therapies and immunotherapy have also been explored in visceral angiosarcomas. Agents targeting VEGF/VEGF receptor pathways and BRAF inhibitors have been reported in selected cases, suggesting potential alternative strategies for advanced PGAS, although evidence remains insufficient to define a standard approach [49].

In the present case, the patient underwent surgical management because the lesion presented as a stenosing ileal process complicated by subocclusive symptoms. Segmental ileal enterectomy was initially performed, followed by a second surgical intervention for postoperative intestinal obstruction caused by stenosis of the ileo-ileal latero-lateral anastomosis and an associated ileocecal inflammatory block. Histopathology confirmed a grade II FNCLCC stenosing ileal angiosarcoma, staged pT1N0Mx. Although the tumor appeared surgically localized at the time of resection, the subsequent clinical course was rapidly unfavorable.

Postoperative chemotherapy was attempted twice in the patient’s local oncology center, but treatment could not be continued because of profound clinical deterioration, circulatory shock, hematological toxicity, and severe sepsis. This inability to tolerate systemic therapy represents a major therapeutic limitation in the present case and may have contributed to the aggressive subsequent evolution. Within a few months, the patient developed suspected tumor recurrence, massive serohemorrhagic ascites, omental infiltration, micronodular mesenteric lesions, worsening anemia, systemic inflammation, and ultimately multiple organ failure.

Therefore, this case illustrates several key therapeutic challenges of small intestinal angiosarcoma: diagnosis is often delayed until surgery, resection may be performed under subocclusive or emergency-like conditions, postoperative complications may occur, and systemic therapy may be difficult to administer because of frailty, sepsis, hematological toxicity, or rapid disease progression. The lack of standardized treatment guidelines further complicates management, emphasizing the need for individualized multidisciplinary decision-making and for reporting additional cases to improve the evidence base for this exceptionally rare malignancy.

### 3.6. Prognosis, Recurrence Pattern and Fatal Evolution

Overall, angiosarcoma is associated with a very poor prognosis, reflecting its highly aggressive biological behavior, marked metastatic potential, and frequent delay in diagnosis. Several studies have suggested that the anatomical site of angiosarcoma may influence clinical outcome [17]. Among the different locations, small intestinal angiosarcoma appears to carry one of the worst prognoses, with outcomes significantly poorer than those reported for angiosarcomas arising in other sites. Most patients with small intestinal angiosarcoma die within a few months after diagnosis, and some succumb during the postoperative period because of complications, tumor progression, or rapid systemic deterioration. Survival beyond one year after diagnosis is considered exceptional in intestinal angiosarcoma [7].

The unfavorable prognosis is related to several factors. First, early symptoms are usually nonspecific, leading to delayed diagnosis and treatment. Second, small intestinal lesions are difficult to access by conventional endoscopy, and imaging findings may mimic inflammatory, obstructive, or adhesive disease. Third, the tumor itself is biologically aggressive, with a high risk of local recurrence, multifocal disease, hemorrhagic complications, peritoneal involvement and distant metastases. Finally, because of the rarity of the disease, therapeutic strategies are not standardized, and systemic treatment is often extrapolated from cutaneous or soft tissue angiosarcoma [8].

The evolution of our patient was consistent with the aggressive course described in the literature. Although the tumor was surgically resected and initially staged as pT1N0Mx, the subsequent clinical course was rapidly unfavorable. Postoperative chemotherapy was attempted but could not be continued because of severe intolerance, including profound deterioration of general condition, circulatory shock, hematological toxicity, and severe sepsis. Within months, the patient developed progressive clinical and biological worsening, massive serohemorrhagic ascites, omental infiltration, micronodular mesenteric lesions, worsening anemia and inflammatory syndrome, and ultimately multiple organ failure.

Although cytological and cytoblock examination of the ascitic fluid did not identify malignant cells, this negative result does not exclude peritoneal tumor involvement, particularly in the setting of hemorrhagic ascites, omental infiltration, mesenteric micronodular lesions, and rapid clinical deterioration. Therefore, the overall evolution was highly suggestive of clinically and radiologically suspected peritoneal progression, rather than histologically or cytologically confirmed tumor recurrence. The patient died on 5 March 2025, less than one year after symptom onset and only a few months after definitive diagnosis, further illustrating the poor prognosis of primary ileal angiosarcoma.

In this context, our case reinforces the need for the early consideration of rare small intestinal malignancies in patients with progressive obstructive symptoms, unexplained anemia or inflammatory syndrome, weight loss, and inconclusive endoscopic findings. Even when complete surgical resection appears feasible, close postoperative surveillance and early multidisciplinary oncologic assessment are essential, although prognosis remains guarded.

### 3.7. Limitations

This case report has several limitations. First, it describes a single patient, and therefore no general conclusions can be drawn regarding the clinical behavior, optimal diagnostic strategy, or therapeutic management of primary ileal angiosarcoma. Given the exceptional rarity of this tumor, interpretation should be made in the context of the limited evidence available from isolated case reports and small literature reviews.

Second, although previous pelvic radiotherapy represents an important etiological clue, a definitive radiation-induced origin cannot be established based on the available data. The patient had received oncological treatment for cervical cancer in medical services from another city, several years before the current admission, and the original radiotherapy records were not available to our team. In addition, the patient did not have the complete historical oncological documentation at presentation. Therefore, data regarding total radiation dose, fractionation schedule, treatment field, and precise anatomical overlap between the irradiated volume and the subsequent ileal tumor site could not be confirmed. In addition, MYC immunohistochemistry or MYC amplification testing could not be performed on the available material, which further limits the ability to support a radiation-associated pathogenesis.

The term “primary ileal angiosarcoma” was used because the ileum represented the only documented site of angiosarcomatous tumor involvement at diagnosis and no other primary angiosarcoma site was identified during the clinical and imaging work-up. However, this classification does not exclude a possible contribution of previous pelvic radiotherapy. Because complete radiotherapy records were unavailable, including total dose, fractionation schedule, treatment field, and precise anatomical overlap with the ileal tumor site, the criteria for confirmed radiation-induced angiosarcoma could not be fully assessed. Therefore, previous pelvic radiotherapy was considered a possible etiological clue rather than definitive proof of causality.

Third, the preoperative diagnosis was limited by the nonspecific clinical presentation and by the restricted accessibility of the small bowel to conventional endoscopic evaluation. Ileocolonoscopy did not identify lesions in the examined terminal ileum, while imaging suggested a stenosing inflammatory process, initially interpreted as possible Crohn’s disease. Thus, the diagnosis could only be established after surgical resection and histopathological/immunohistochemical examination.

Fourth, complete details regarding the oncological treatment attempted after surgery were not available. Although chemotherapy was reportedly initiated twice, the exact regimen, doses, number of cycles, and timing in relation to postoperative recovery were not fully documented. This limits the ability to evaluate the potential efficacy or toxicity profile of systemic therapy in this patient.

Fifth, although the patient subsequently developed massive serohemorrhagic ascites, omental infiltration, micronodular mesenteric lesions, progressive anemia, inflammatory syndrome, and rapid clinical deterioration, cytological and cytoblock examination of the ascitic fluid did not identify malignant cells. Therefore, tumor recurrence or peritoneal involvement was suspected based on the clinical and radiological course, but it was not cytologically confirmed. Also, no autopsy was performed, because the patient’s family did not consent to postmortem examination. Therefore, the clinically and radiologically suspected late peritoneal progression could not be confirmed by autopsy, which represents an additional limitation of the case. Finally, the rapid unfavorable evolution and death of the patient limited long-term follow-up. As a result, the natural history of the disease after surgery, the potential impact of systemic therapy, and the precise pattern of recurrence could not be fully characterized. Despite these limitations, the case remains clinically relevant because it highlights the diagnostic difficulty, possible radiation-associated background, aggressive behavior, and poor prognosis of primary ileal angiosarcoma.

## 4. Conclusions

Primary ileal angiosarcoma is an exceptionally rare and highly aggressive malignancy, often associated with nonspecific clinical manifestations and delayed diagnosis. The present case highlights the diagnostic difficulty of this entity, which initially mimicked functional bowel disease, postoperative adhesive syndrome, and later stenosing Crohn’s disease. The absence of endoscopic lesions in the examined terminal ileum further complicated the diagnostic pathway, while the definitive diagnosis was established only after surgical resection and histopathological/immunohistochemical confirmation.

This case is particularly relevant because the tumor developed in a patient with previous pelvic surgery and radiotherapy for cervical cancer. Although a radiation-induced origin cannot be confirmed in the absence of detailed dosimetric data and precise radiation field correlation, previous pelvic radiotherapy may represent a clinically important etiological clue and should be considered when evaluating progressive stenosing small-bowel lesions in long-term survivors of gynecological malignancies.

Despite surgical treatment, the clinical course was rapidly unfavorable, with clinically and radiologically suspected peritoneal progression, massive serohemorrhagic ascites, systemic deterioration, and death within a few months after diagnosis. Therefore, primary small intestinal angiosarcoma should be considered, although rarely, in the differential diagnosis of progressive obstructive small-bowel symptoms, especially when associated with weight loss, anemia, inflammatory syndrome, inconclusive endoscopy, and a history of pelvic radiotherapy. Early multidisciplinary assessment, surgical management when feasible, histopathological confirmation, and close postoperative surveillance are essential, although prognosis remains poor due to the aggressive nature of this malignancy and the lack of standardized therapeutic guidelines.

## Figures and Tables

**Figure 1:**
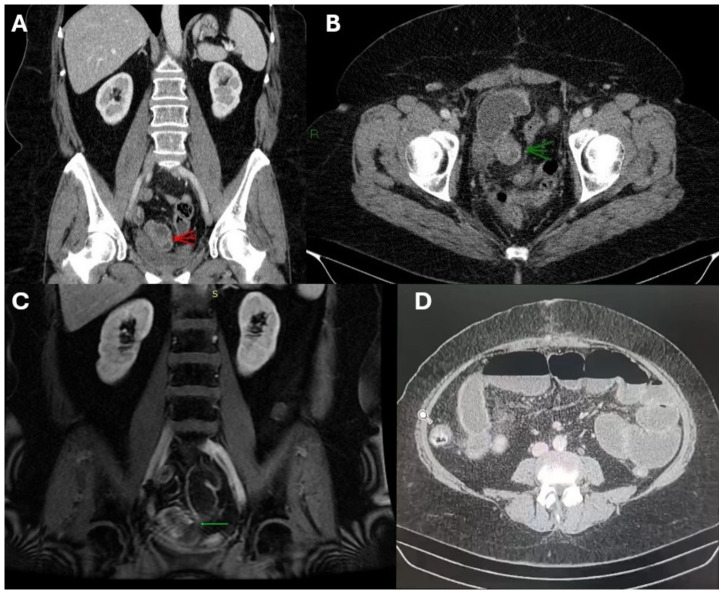
Radiological findings during the diagnostic and postoperative course. (**A**) Contrast-enhanced abdomino-pelvic CT demonstrating a fluid-filled dilated small-bowel loop in the pelvis. (**B**) Native abdomino-pelvic CT showing fluid-filled dilated small-bowel loops. (**C**) Entero-MRI showing right parasagittal pelvic circumferential gadolinium-enhancing ileal wall thickening, associated with stenosis and upstream small-bowel dilation, initially suggestive of stenosing Crohn’s disease. (**D**) Postoperative day 4 abdomino-pelvic CT showing the collapse of the ileal loop distal to the latero-lateral anastomosis, with fluid-filled dilation of the upstream small bowel. The colon was collapsed, and no intra-abdominal collections were identified.

**Figure 2:**
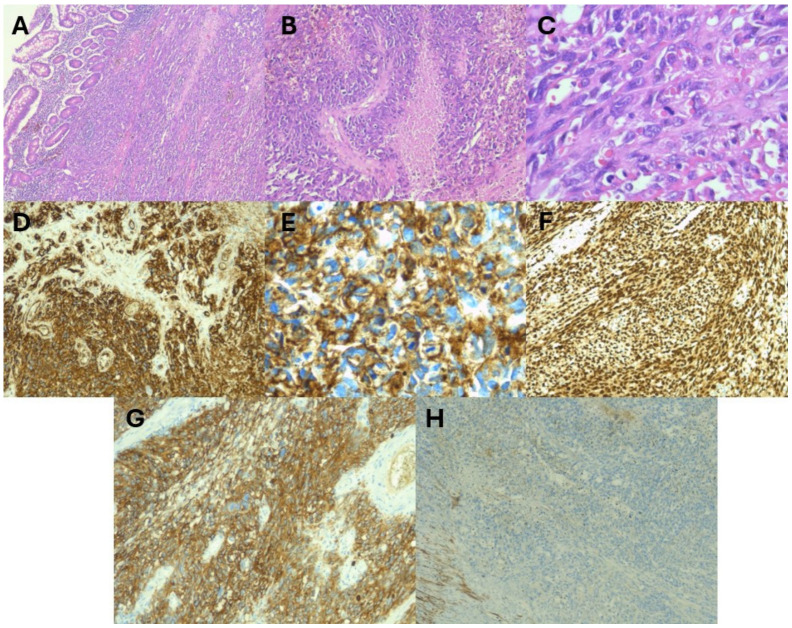
Histopathological and immunohistochemical findings of ileal angiosarcoma. (**A**) A low-power hematoxylin and eosin section showing tumor infiltration within the ileal wall (H&E, ×5). (**B**) A hematoxylin and eosin section demonstrating neoplastic proliferation associated with tumor necrosis (H&E, ×10). (**C**) A high-power hematoxylin and eosin section showing mitotic activity within the tumor proliferation (H&E, ×40). (**D**) Immunohistochemical staining demonstrating CD31 positivity in the neoplastic cells, supporting endothelial differentiation (CD31, ×10). (**E**) Immunohistochemical staining showing CD34 positivity in part of the neoplastic vascular proliferation, supporting endothelial differentiation (CD34, ×40). (**F**) Immunohistochemical staining demonstrating nuclear ERG positivity in the tumor cells, confirming endothelial lineage (ERG, ×10). (**G**) c-KIT staining without significant tumor cell expression, arguing against gastrointestinal stromal tumor (c-KIT, ×10). (**H**) DOG1 staining without significant tumor cell expression, further supporting the exclusion of gastrointestinal stromal tumor (DOG1, ×5).

**Table 1:** Selected reported cases of primary angiosarcoma with ileal or terminal ileal involvement and comparison with the present case.

Reference	Age/Sex	Tumor Location	Predisposing Factor/Oncologic History	Main Clinical Presentation	Outcome/Prognosis
Chen et al. [27]	F/66	Terminal ileum	Radiotherapy for endometrioid adenocarcinoma/ovary; 60 Gy; latency 8 years	Abdominal pain, nausea, vomiting	Died of disease at 14 months
Nanus et al. [28]	F/42	Distal ileum	Radiotherapy for ovarian dysgerminoma; 48 Gy; latency 16 years	Perforated distal ileum	Died of disease at 36 months
Suzuki et al. [29]	F/61	Terminal ileum	Radiotherapy for squamous cell carcinoma of the uterine cervix; dose not available; latency 20 years	Intestinal perforation	Died of disease at 12 months
Aitola et al. [30]	F/50	Terminal ileum	Radiotherapy for stage I endometrial adenocarcinoma; 55.6 Gy; latency 14 years	Intestinal obstruction	Alive with disease at 21 months
Policarpio-Nicolas et al. [31]	F/51	Terminal ileum	Radiotherapy for stage IIB endometrioid uterine adenocarcinoma; 50 Gy; latency 9 years	Decreased appetite, abdominal pain, increasing abdominal girth	Died of disease at 10 months
Present case	F/54	Distal/terminal ileum	Previous cervical squamous cell carcinoma treated with Wertheim-type surgery, chemotherapy, and external pelvic radiotherapy; latency approximately 8 years	Progressive colicky abdominal pain, constipation, bilio-enteric vomiting, anemia, inflammatory syndrome, 13 kg weight loss; initially mimicking stenosing Crohn’s disease	Rapid unfavorable evolution with suspected peritoneal progression, massive serohemorrhagic ascites, multiple organ failure, and death

## Data Availability

The data presented in this case report are not publicly available due to patient privacy and confidentiality considerations. Relevant clinical, imaging, histopathological, and laboratory data supporting the findings of this report are included within the article.

## Abbreviations

The following abbreviations are used in this manuscript:

CT	Computed tomography
MRI	Magnetic resonance imaging
PET-CT	Positron emission tomography–computed tomography
ESR	Erythrocyte sedimentation rate
CRP	C-reactive protein
PCR	Polymerase chain reaction
CFU	Colony-forming units
PGAS	Primary gastrointestinal angiosarcoma
FNCLCC	Fédération Nationale des Centres de Lutte Contre le Cancer
TNM	Tumor–node–metastasis
pT1N0Mx	Pathological tumor stage T1, node-negative, distant metastasis not assessed
R0	Complete microscopic tumor resection with negative margins
CD31	Cluster of differentiation 31
CD34	Cluster of differentiation 34
ERG	ETS-related gene
c-KIT	CD117 proto-oncogene receptor tyrosine kinase
DOG1	Discovered on gastrointestinal stromal tumors 1
FLI-1	Friend leukemia integration 1 transcription factor
VEGF	Vascular endothelial growth factor
UEA	Ulex europaeus agglutinin
BRAF	B-Raf proto-oncogene
H&E	Hematoxylin and eosin
Gy	Gray
GI	Gastrointestinal
